# Molecular Surface Quantification of Multifunctionalized Gold Nanoparticles Using UV–Visible Absorption Spectroscopy Deconvolution

**DOI:** 10.1021/acs.analchem.3c01649

**Published:** 2023-08-25

**Authors:** Jordan C. Potts, Akhil Jain, David B. Amabilino, Frankie J. Rawson, Lluïsa Pérez-García

**Affiliations:** † Division of Advanced Materials and Healthcare Technologies, School of Pharmacy, University of Nottingham, Nottingham NG7 2RD, U.K.; ‡ Bioelectronics Laboratory, Division of Regenerative Medicine and Cellular Therapies, School of Pharmacy, University of Nottingham, Biodiscovery Institute, Nottingham NG7 2RD, U.K.; § Institut de Ciència de Materials de Barcelona (ICMAB), CSIC, Carrer dels Til·lers, Campus Universitari, 08193 Cerdanyola del Vallès, Catalunya, Spain; ∥ Departament de Farmacologia, Toxicologia i Química Terapèutica, Facultat de Farmàcia i Ciències de l’Alimentació, Universitat de Barcelona, 08028 Barcelona, Spain; ⊥ Institut de Nanociència i Nanotecnologia UB (IN2UB), Universitat de Barcelona, 08028 Barcelona, Spain

## Abstract

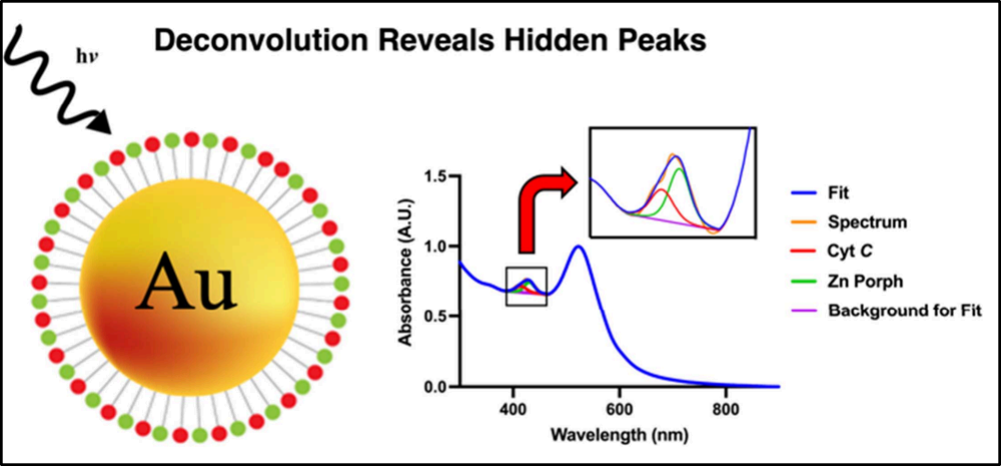

Multifunctional gold nanoparticles (AuNPs) are of great interest, owing to their vast potential for use in many areas including sensing, imaging, delivery, and medicine. A key factor in determining the biological activity of multifunctional AuNPs is the quantification of surface conjugated molecules. There has been a lack of accurate methods to determine this for multifunctionalized AuNPs. We address this limitation by using a new method based on the deconvolution and Levenberg–Marquardt algorithm fitting of UV–visible absorption spectrum to calculate the precise concentration and number of cytochrome *C* (Cyt *C*) and zinc porphyrin (Zn Porph) bound to each multifunctional AuNP. Dynamic light scattering (DLS) and zeta potential measurements were used to confirm the functionalization of AuNPs with Cyt *C* and Zn Porph. Transmission electron microscopy (TEM) was used in conjunction with UV–visible absorption spectroscopy and DLS to identify the AuNP size and confirm that no aggregation had taken place after functionalization. Despite the overlapping absorption bands of Cyt *C* and Zn Porph, this method was able to reveal a precise concentration and number of Cyt *C* and Zn Porph molecules attached per AuNP. Furthermore, using this method, we were able to identify unconjugated molecules, suggesting the need for further purification of the sample. This guide provides a simple and effective method to quickly quantify molecules bound to AuNPs, giving users valuable information, especially for applications in drug delivery and biosensors.

AuNPs have gained broad research interest due to their attractive properties such as optical, chemical inertness, facile synthesis, and surface chemistry. These properties make AuNPs ideal for biological and medical applications, including use in biosensors, genomics, targeted delivery of drugs, DNA and antigens, optical bioimaging, and clinical chemistry, to name but a few.^1−4^

The ability to functionalize AuNPs with molecules has led to an interest in designing more complex systems with multiple molecule types conjugated to each AuNP, known as multifunctionalized AuNPs. These multifunctionalized AuNPs have a significant advantage over AuNPs conjugated with just one type of molecule as they can be used for broader applications.^5^ An example of multifunctionalized AuNPs is in drug delivery systems, where a targeting agent and drug are conjugated to the surface of the particle to direct the effect of the drug to a specific cell type or region.^6−8^ The concentration of conjugated molecules on the surface of the AuNPs is an important factor that determines its nanobiointeractions and, eventually, its biological function. In pharmaceutical applications, the concentration of the conjugated molecules is a significant factor in modulating drug dose thresholds.

In biosensing, the minimum concentration of analyte that can be detected is known as its limit of detection or sensitivity.^9^ Working below the limit of detection will make the results inaccurate and invalid. Consequently, there is a need to develop a simple, robust, and fast analytical method to facilitate surface quantification of AuNPs functionalized with more than one molecule. We have recently reported on the development of a multifunctional AuNP system functionalized with Cyt *C* for cell-induced apoptosis.^10^ Cyt *C* is a biomolecule of interest due to its redox properties that make it useful for biosensing as well as its ability to induce apoptosis, lending it to applications in drug delivery.^11−13^ UV–visible absorption spectroscopy was used to quantitate the presence of Cyt *C* on AuNPs functionalized with ligands of varying electrostatic charge.^10^ Also, determining the presence of monolayers or multilayers was important to know when considering the concentration of drugs or biomolecules conjugated to AuNPs as a multilayer may interfere with their function, leading to an inaccurate representation of drug/biomolecule delivery or activity.

Biomolecule concentration on functionalized AuNPs can be assessed using photometric and fluorometric assays to confirm conjugation. Intensity changes in these assays enable calculation of conjugated molecule concentration.^14^ Alternatively, advanced techniques like MALDI-TOF mass spectrometry can be utilized for protein quantification.^15^ Analyzing multifunctionalized AuNPs’ concentration of each functional molecule presents challenges due to spectral complexities, which can yield inaccurate results, particularly when absorption spectra of conjugated molecules overlap.

To address the issue of quantifying molecules attached to AuNPs, we have introduced a new analysis protocol for molecular quantification of AuNP functionalized with a Zn Porph and biomolecule Cyt *C*. This lets us accurately measure the concentration of various molecules on the AuNPs’ surface. We began by producing AuNPs of 20 nm and 50 nm in diameters and then functionalized them with Zn Porph and Cyt *C*. Due to the overlapping absorption spectra of Zn Porph and Cyt *C*, it was challenging to determine the exact concentration of the molecules attached to the AuNPs. However, we applied CASA-XPS to separate the convoluted absorption spectra of both conjugated molecules allowing for their quantification. As a result, we could discern the peak heights for each molecule, allowing us to calculate their respective concentrations. This method can be used as a guide by researchers to tailor conjugation parameters for obtaining multifunctionalized AuNPs for the desired application.

## Experimental Section

The key method developed is described below. The Supporting Information includes nanoparticle fabrication and physical characterization methods used to characterize them through TEM, DLS, and zeta potential can be found in the Supporting Information. Ultraviolet–visible (UV–visible absorption) absorption spectra were obtained using a Varian Cary 50 bio-UV–visible absorption spectrophotometer. Deconvolution of the UV–visible absorption spectrum confirming the binding of Cyt *C* and Zn Porph was performed using CASA-XPS. The absorption spectra of the chosen samples were imported into CASA-XPS as text files. The graph was then plotted, and the *x*-axis was changed to start with the smallest number near the origin. The region of interest was then selected using the regions tool, and then the background setting was changed to “linear” as it closely represents how the spectra would look without the conjugated molecules. The convoluted peaks in the spectra were then found using the components tab, by adding the components that were thought to be in the region. The components were then fitted to the spectra using Levenberg–Marquardt algorithm LN (LN-MIE-Gans) fitting. The resulting spectra data were copied to Microsoft Excel before being added to GraphPad Prism 9 for analysis.

## Results and Discussion

**Figure 1:**
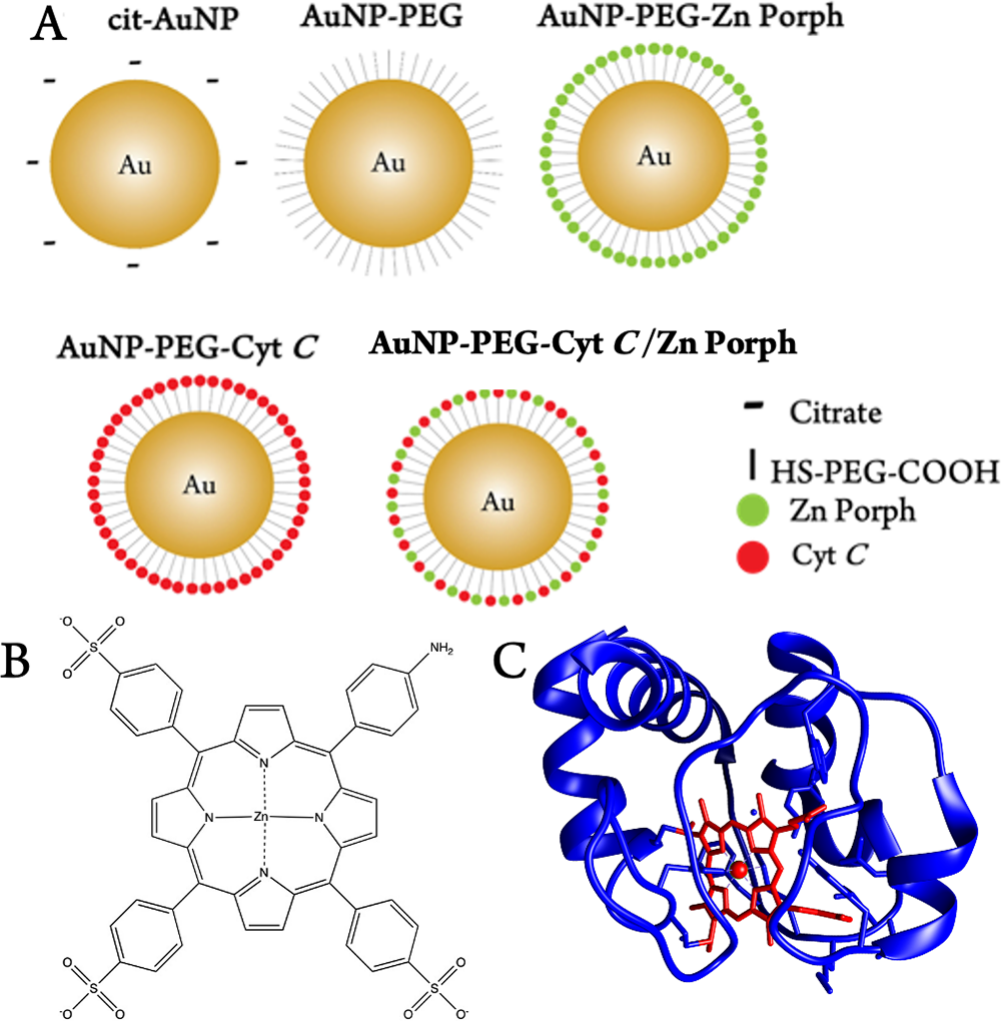
Schematic representation of as-synthesized and thiol-PEG-carboxylic, cytochrome *C* (Cyt *C*) and zinc(II) 5-(4-aminophenyl)-10,15,20-tris(4-sulfonatophenyl)- porphyrin (Zn Porph) functionalized cit-AuNPs (A). Structure of Zn Porph (B). 3D structure crystal of Cyt *C* made from PDB (1 HRC) using Chimera (version 1.16) with blue representing amino acid residues and red representing the Heme ring (C).

The freshly prepared cit-AuNPs were functionalized with the HS-PEG-COOH to produce AuNP-PEG samples. Carbodiimide coupling chemistry was then employed to covalently conjugate Cyt *C* and Zn Porph to yield the AuNP-PEG-Cyt *C*/Zn Porph sample. Schematic representations of cit-AuNP, AuNP-PEG, AuNP-PEG-Zn Porph, AuNP-PEG-Cyt *C*, and AuNP-PEG-Cyt *C*/Zn Porph are shown in Figure 1A. The chemical structure of Zn Porph is represented in Figure 1B. The 3D structure of Cyt *C* is shown in Figure 1C with the amino acid residues represented in blue and the Heme porphyrin ring represented in red.

The physical characterization of the multifunctionalized AuNPs (AuNP-PEG-Cyt *C*/Zn Porph) was conducted to enable quantification of the conjugated molecules, Cyt *C* and Zn Porph, using dynamic light scattering (DLS) (Figure S1A), zeta potential (Figure S1B), transmission electron microscopy (TEM) (Figure S1C), and UV–visible absorption spectroscopy (Figure S1D). To summarize the findings, DLS showed that the hydrodynamic diameter (hd) increased from 19.6 ± 1.0 nm (polydispersity index (PDI) = 0.301) in cit-AuNPs to 33.54 ± 0.50 nm (PDI = 0.297) in AuNP-PEG and 38.9 ± 1.1 nm (PDI = 0.388) for AuNP-PEG-Cyt *C*/Zn Porph (Figure S1A). The zeta potential measurement of cit-AuNPs was recorded to be −36.77 ± 2.33 mV, which is a slightly lower value of −30.77 ± 1.4 mV for AuNP-PEG. Cyt *C* is known to have a positive zeta potential measurement, which is exhibited on the AuNP-PEG-Cyt *C* samples at +14.7 ± 0.9 mV, confirming the successful conjugation of Cyt *C* on PEG. The functionalization process does not cause any aggregation (Figure S1C). Furthermore, the cumulative frequency graph (inset in Figure S1C) reveals a mean average diameter of AuNP-PEG-Cyt *C*/Zn Porph samples to be around 19.15 ± 2.12 nm (Gaussian fit).

The conjugation of Cyt *C* and Zn Porph to AuNPs was further confirmed using UV–visible absorption spectroscopy, which revealed changes in the surface chemistry (Figure S1D). The full-width at half-maximum (fwhm) of the surface plasmon resonance (SPR) peak increased in all the functionalized AuNP samples, suggesting an increase in polydispersity. Additionally, there was an increase in intensity between 650 and 700 nm in AuNP-PEG-Cyt *C* due to small aggregates from protein-nanoparticle interactions. The UV–visible absorption spectra showed the Soret band peaks of Cyt *C* and Zn Porph overlapped in multifunctional AuNP-PEG-Cyt *C*/Zn Porph samples, making accurate peak determination difficult (Figure S1D). This highlights the need for deconvolution of the overlapping spectra for correct quantification and is supported by the spectra of Cyt *C* and Zn Porph free in solution, resulting in convoluted Soret band peaks (Figure S2A-E). A detailed discussion of the characterization is presented in the Supporting Information (SI), suggesting successful conjugation.

**Figure 2:**
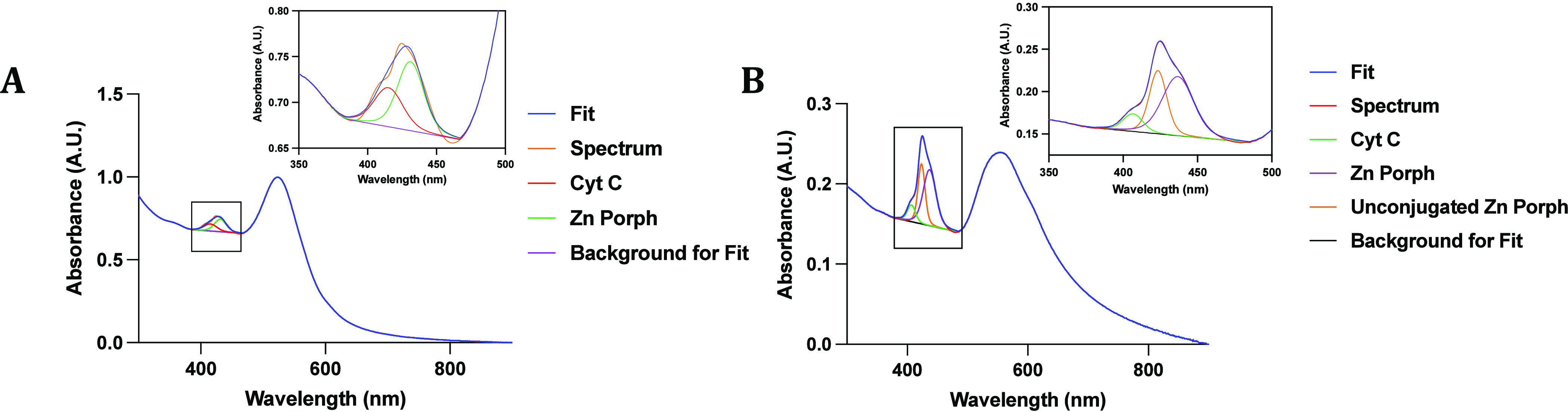
Soret band in the ultraviolet–visible spectra of AuNP-PEG-Cyt *C*/Zn Porph was deconvoluted to identify two fitted components in AuNP-PEG-Cyt *C*/Zn-Porph samples (A) and three fitted components in AuNP-PEG-Cyt *C*/Zn-Porph-S2 (B). I propose UV–vis deconvolution to identify hidden peaks and unbound molecules in multifunctional AuNPs. (A) Two fitted component deconvolutions of soret band peak in AuNP-PEG-Cyt C/Zn-Porph samples. (B) Three fitted component deconvolution in AuNP-PEG-Cyt C/Zn-Porph-S2 sample.

The spectral overlap of the Soret band of Cyt *C* and Zn Porph (Figure S2A) prevents the identification of individual Soret peak heights in AuNP-PEG-Cyt *C*/Zn Porph. Therefore, deconvolution of the Soret band of AuNP-PEG-Cyt *C*/Zn Porph was conducted to accurately find the contributions of Cyt *C* and Zn Porph in the functionalized AuNP samples (Figure 2A). The Soret band was selected as a region of interest in CASA-XPS, and two components (the first one for Cyt *C* and the second for Zn Porph) were selected to be fitted in the AuNP-PEG-Cyt *C*/Zn Porph absorption spectrum shown in Figure 2A. These were located at 415 nm, attributed to Cyt *C*, and at 430 nm for Zn Porph. The components were fitted using the Levenberg–Marquardt algorithm fitting, which resulted in a good fit with a residual standard deviation (RSD) of 0.006. Therefore, the good fit confirms that only two components are located within the peak and that the deconvolution can accurately determine the heights of the hidden peaks.

To identify if the deconvolution method would work in another AuNPs system with larger Soret band peaks, Cyt *C* and Zn Porph were functionalized on AuNP-PEG particles, namely, AuNP-PEG-Cyt *C*/Zn Porph-S2. This sample was prepared using the same concentrations of Cyt *C* and Zn Porph as in AuNP-PEG-Cyt *C*/Zn Porph. However, the AuNP concentration was attenuated from 1.21 to 0.26 nM (Figure 2B). The resulting Soret band in the UV–vis spectrum was characterized by flanking shoulders and manifested a deviation in form when contrasted with the Soret band illustrated in Figure 2A. In this case, fitting the deconvoluted spectrum with just two components resulted in a large RSD, suggesting the presence of another component. Therefore, the third component at 423 nm, which corresponds to the absorption peak of free unconjugated Zn Porph (Figure S2A), was added to improve the fit. The fitting resulted in an RSD of 0.002 (Figure 2B). A third component supposes that free/unconjugated Zn Porph is still present in the solution together with Zn Porph conjugated to AuNPs. Therefore, suggesting further purification of the sample is required. Interestingly, the formation of Zn Porph J-aggregates can also be identified using this method, which is of great importance as the fluorescence properties of porphyrins depend on their aggregation state and local environment.^16,17^ This was also observed when deconvoluting the UV–Vis absorption spectra of free Cyt *C* OX and Zn Porph in water (Figure S2D). Figure 2B also shows a wider SPR peak that appears to have a peak around 553 nm. This is due to the higher concentration of Zn Porph and Cyt *C* to AuNPs in the sample; therefore, Cyt *C*’s peak at 500–600 nm and the Q-bands of Zn Porph from 550 to 650 nm influence the spectrum more than that shown in Figure 2A. Further characterization of the sample in Figure 2B by TEM indicated that there was no aggregation of the AuNPs (Figure S3A) as the diameter was determined to be 19.3 ± 2.5 nm; however, the DLS average diameter (Figure S3B) shows a hd of 43.0 ± 0.9 nm (PDI = 0.353). This is a result of aggregation of a small percentage of the AuNPs in the sample as the intensities of the aggregates in the DLS profile show (Figure S3C).

The identification of unbound molecules in AuNP-PEG-Cyt *C*/Zn Porph-S2 using the deconvolution method is essential for ensuring the accuracy of the concertation of molecules bound to AuNPs. Excess unbound molecules would influence other analytical techniques and experiments; therefore, it is important to remove the unbound molecules, once identified. The deconvolution method should be used regularly together with UV–vis for accurate quantification of bound molecules, especially in applications such as drug delivery, where UV–visible absorption is often used to quantify surface-conjugated molecules. To calculate the concentration of each component (Cyt *C* and Zn Porph) attached to AuNP-PEG-Cyt *C*/Zn Porph (Figure 2A) and AuNP-PEG-Cyt *C*/Zn Porph-S2 (Figure 2B) samples, it was essential to first calculate the concentration of AuNPs in each sample.

To calculate the AuNP concentration, the minimum absorbance values ∼470–480 nm and between the SPR and peaks of the conjugate molecules were used to calculate AuNP concentration. These absorbance values were then divided by the extinction coefficient of 20 nm of AuNPs (5.41 × 10^8^ M^–1^ cm^–1^) to obtain the concentration of the AuNPs in the samples. By using the above method, a concentration of 1.21 and 0.26 nM was obtained for AuNPs in AuNP-PEG-Cyt *C*/Zn Porph (Figure 2A) and AuNP-PEG-Cyt *C*/Zn Porph-S2 (Figure 2B) samples, respectively. These concentration values were then multiplied by the Avogadro’s constant (6.022 ×́ 10^23^) to obtained 7.30 × 10^14^ AuNP/L (AuNP-PEG-Cyt *C*/Zn Porph) and 1.55 × 10^14^ AuNP/L (AuNP-PEG-Cyt *C*/Zn Porph-S2).

Next, for the determination of the concentration and number of Cyt *C* and Zn Porph per AuNP, we corrected the actual absorbance values by subtracting the background absorbance readings from the height of the deconvoluted peaks at 414 nm. The obtained background corrected absorbance values of 0.04 and 0.02 A.U. for AuNP-PEG-Cyt *C*/Zn Porph and AuNP-PEG-Cyt *C*/Zn Porph-S2, respectively, were then divided by the extinction coefficients of Cyt *C* (101600 M^–1^ cm^–1^, literature value) and Zn Porph (57940 M^–1^ cm^–1^, calculated from a concentration curve in Figure S4) to obtain the concentration of these molecules.^18^ The detailed calculations for obtaining the concentrations of the AuNP-PEG-Cyt *C*/Zn Porph and AuNP-PEG-Cyt *C*/Zn Porph-S2 samples (shown in Figure S3A,B) are presented in Table S1. The concentration of Cyt *C* in the AuNP-PEG-Cyt *C*/Zn Porph sample (Figure 2A) was calculated to be 0.42 μM, which corresponds to 2.53 × 10^17^ Cyt *C* molecules/L. Similarly, the concentration of Zn Porph in AuNP-PEG-Cyt *C*/Zn Porph sample (Figure 2A) was calculated to be 1.3 μM, with a subsequent 7.83 × 10^17^ Zn Porph molecules/L. By dividing the number of molecules of Cyt *C* and Zn Porph per liter by the number of AuNPs per liter, the number of each molecule bound to a single AuNP was calculated to be 346 and 1073, respectively. Similarly, the concentration of AuNP in the AuNP-PEG-Cyt *C*/Zn Porph-S2 sample (Figure 2B) was calculated to be 0.26 nM and 1.55 × 10^14^ AuNPs/L. The concentrations of Cyt *C* and Zn Porph were calculated to be 0.40 and 1.16 μM, equating to 2.4 × 10^17^ and 6.99 × 10^17^ molecules/L, respectively. Thus, the number of Cyt *C* and Zn Porph bound to each AuNP was calculated to be 1545 and 4500, respectively. However, the deconvolution analysis revealed a free Zn Porph peak, which is a result of unconjugated and excess Zn Porph in the AuNP-PEG-Cyt *C*/Zn Porph-S2 sample. It is important to note this peak corresponding to free Zn-Porph was not obtained after the deconvolution of the UV–vis spectrum of the AuNP-PEG-Cyt *C*/Zn Porph sample. The concentration of this free and unconjugated Zn Porph in the AuNP-PEG-Cyt *C*/Zn Porph-S2 sample was calculated to be 1.11 μM. This can be further implied from the number of conjugated molecules/AuNP in the AuNP-PEG-Cyt *C*/Zn Porph-S2 sample, which is over fourfold compared to the number of conjugated molecules in the AuNP-PEG-Cyt *C*/Zn Porph sample. This confirms that the free and unconjugated Zn Porph in the mixture hinders the accurate determination of the number of molecules bound to each AuNP.

Therefore, the deconvolution of UV–vis peaks on AuNP samples functionalized with more than one molecule provides users with important information about the concentration and number of conjugated molecules. This method could also provide insight on the quality and percentage coverage of a self-assembled monolayer of molecules on a nanoscale object, which is essential for assessing the performance of multifunctional systems such as nanosensors and nanomedicines.

To prove the robustness and reproducibility of this method, another example of Soret band deconvolution was conducted on the sample with a high degree of spectral overlap in 50 nm AuNPs (Figure S5A). The Soret band of Cyt *C* and a nonmetalated porphyrin (Porph, structure in Figure S5B) appears as a single peak when functionalized to 50 nm cit-AuNPs with HS-PEG-COOH (SPR at 560 nm). The DLS shows that the hd of 50 nm cit-AuNPs grows from 49.21 ± 0.81 nm (PDI = 0.263) to 65.64 ± 1.28 nm (PDI = 0.236) with the conjugation of HS-PEG-COOH 2 kDa and then 172.2 ± 5.00 nm (PDI = 0.239) with the addition of HS-PEG-COOH and Cyt *C* (Figure S5C). The DLS results suggest that the Cyt *C* is arranged in a multilayer around the 50 nm AuNPs as the TEM images (Figure S5D), and cumulative frequency distribution produced from analyzing the diameter of 163 AuNPs (Figure S5E) had an average diameter of 35.42 ± 4.33 nm and therefore showed no sign of aggregation. The maximum Soret band absorbance of Porph at 415 nm shows a greater overlap with reduced Cyt *C* (*l*_max_ = 415 nm) (Figure S5F) compared to that of Zn Porph at 423 nm; therefore, individual component concentrations cannot be calculated from the convoluted peak. The deconvolution enables the concentrations of Cyt *C* and Porph mixed free in solution (Figure S5G) to be calculated as 2.12 and 2.65 mM, respectively, closely matching the 2.5 mM theoretical concentration of the prepared solutions. In AuNP-PEG-Cyt *C*/Porph the concentrations of 50 nm AuNPs (extinction coefficient^18^ = 9.92 × 10^9^), Cyt *C*, and Porph were calculated to be 9.6 pM, 0.11 mM, and 0.17 mM, respectively. The coverage of Cyt *C* and Porph per AuNP was 23259 and 35168, respectively (Table S1). The higher number of molecules per AuNP is due to the lower concentration of AuNPs in the sample and their larger diameter.

Deconvolution was not conducted on the peak between 500 and 600 nm, as this region is attributed to the SPR peak of the AuNPs. The LN-MIE-Gans model fitting taken from the literature of 20 nm AuNPs evidences this (Figure S5A), as it can predict the radius of the AuNPs based on the fit of the model to the spectra (Figure S5B).^19^ For the spectrum of 20 nm cit-AuNPs, the radius is predicted to be 10 nm with a resultant predicted diameter of 20 nm. The predicted diameter correlates with those calculated previously from the TEM at 19.15 ± 2.12 nm and DLS at 19.57 ± 1.03 nm (cit-AuNPs); therefore, it can be assumed that the only region of interest for deconvolution in these examples is within the Soret band region.

## Conclusion

In conclusion, we have established a simple, robust, and reproducible method to accurately quantify the concentration and the exact number of molecules that are bound to AuNPs. Deconvolution of UV–visible absorption spectra that can be used together with DLS, zeta potential, and TEM is highly recommended to build an accurate understanding of the quantity and nature of conjugated molecules on multifunctionalized AuNPs. This method of accurately determining the concentration of each molecule bound to multifunctionalized AuNPs could be used by researchers from various disciplines, for instance, to tailor the material surface and binding concentration based on the desired application without misinterpreting the binding concentration.

## Supporting Information Available

Complete methodologies; UV–visible absorption spectra of free Cyt *C* and Zn Porph, both individually and in combination (Figure S2); physicochemical attributes of 20 cit-AuNPs (Figure S3); detailed calculation to obtain the concentration and number of Cyt *C*, Zn Porph, and AuNPs (Table S1); calibration curve of Zn Porph to determine its extinction coefficient (Figure S4); physicochemical characteristics of 50 nm functionalized AuNPs are displayed (Figure S5), alongside an example of LN-MIE-Gans model fitting (Figure S5) (PDF)

## References

[ref1] Dykman L. A.; Khlebtsov N. G. Gold nanoparticles in biology and medicine: recent advances and prospects. Acta naturae 2011, 3 (2), 34–55. 10.32607/20758251-2011-3-2-34-55. 22649683 PMC3347577

[ref2] Nejati K.; Dadashpour M.; Gharibi T.; Mellatyar H.; Akbarzadeh A. Biomedical Applications of Functionalized Gold Nanoparticles: A Review. Journal of Cluster Science 2022, 33 (1), 1–16. 10.1007/s10876-020-01955-9.

[ref3] Duan L.; Ouyang K.; Xu X.; Xu L.; Wen C.; Zhou X.; Qin Z.; Xu Z.; Sun W.; Liang Y. Nanoparticle Delivery of CRISPR/Cas9 for Genome Editing. Front Genet 2021, 12, 673286–673286. 10.3389/fgene.2021.673286. 34054927 PMC8149999

[ref4] Trabbic K. R.; Kleski K. A.; Barchi J. J. Stable Gold-Nanoparticle-Based Vaccine for the Targeted Delivery of Tumor-Associated Glycopeptide Antigens. ACS Bio & Med. Chem. Au 2021, 1 (1), 31–43. 10.1021/acsbiomedchemau.1c00021. PMC8675876 34927166

[ref5] Mieszawska A. J.; Mulder W. J. M.; Fayad Z. A.; Cormode D. P. Multifunctional gold nanoparticles for diagnosis and therapy of disease. Mol. Pharmaceutics 2013, 10 (3), 831–847. 10.1021/mp3005885. PMC3593826 23360440

[ref6] Hosta-Rigau L.; Olmedo I.; Arbiol J.; Cruz L. J.; Kogan M. J.; Albericio F. Multifunctionalized Gold Nanoparticles with Peptides Targeted to Gastrin-Releasing Peptide Receptor of a Tumor Cell Line. Bioconjug Chem. 2010, 21 (6), 1070–1078. 10.1021/bc1000164. 20476781

[ref7] Mohamed M. S.; Veeranarayanan S.; Poulose A. C.; Nagaoka Y.; Minegishi H.; Yoshida Y.; Maekawa T.; Kumar D. S. Type 1 ribotoxin-curcin conjugated biogenic gold nanoparticles for a multimodal therapeutic approach towards brain cancer. Biochimica et Biophysica Acta (BBA) - General Subjects 2014, 1840 (6), 1657–1669. 10.1016/j.bbagen.2013.12.020. 24361614

[ref8] Taghdisi S. M.; Danesh N. M.; Lavaee P.; Emrani A. S.; Hassanabad K. Y.; Ramezani M.; Abnous K. Double targeting, controlled release and reversible delivery of daunorubicin to cancer cells by polyvalent aptamers-modified gold nanoparticles. Mater. Sci. Eng. C Mater. Biol. Appl. 2016, 61, 753–61. 10.1016/j.msec.2016.01.009. 26838906

[ref9] Bhalla N.; Jolly P.; Formisano N.; Estrela P. Introduction to biosensors. Essays Biochem 2016, 60 (1), 1–8. 10.1042/EBC20150001. 27365030 PMC4986445

[ref10] Jain A.; Trindade G. F.; Hicks J. M.; Potts J. C.; Rahman R.; Hague R. J. M.; Amabilino D. B.; Pérez-García L.; Rawson F. J. Modulating the biological function of protein by tailoring the adsorption orientation on nanoparticles. J. Colloid Interface Sci. 2021, 587, 150–161. 10.1016/j.jcis.2020.12.025. 33360888

[ref11] Manickam P.; Kaushik A.; Karunakaran C.; Bhansali S. Recent advances in cytochrome c biosensing technologies. Biosens. Bioelectron. 2017, 87, 654–668. 10.1016/j.bios.2016.09.013. 27619529

[ref12] Guo C.; Wang J.; Chen X.; Li Y.; Wu L.; Zhang J.; Tao C.-a. Construction of a Biosensor Based on a Combination of Cytochrome c, Graphene, and Gold Nanoparticles. Sensors 2019, 19 (1), 40 10.3390/s19010040. PMC6339241 30583520

[ref13] Delinois L. J.; De León-Vélez O.; Vázquez-Medina A.; Vélez-Cabrera A.; Marrero-Sánchez A.; Nieves-Escobar C.; Alfonso-Cano D.; Caraballo-Rodríguez D.; Rodriguez-Ortiz J.; Acosta-Mercado J.; Benjamín-Rivera J. A.; González-González K.; Fernández-Adorno K.; Santiago-Pagán L.; Delgado-Vergara R.; Torres-Ávila X.; Maser-Figueroa A.; Grajales-Avilés G.; Méndez G. I. M.; Santiago-Pagán J.; Nieves-Santiago M.; Álvarez-Carrillo V.; Griebenow K.; Tinoco A. D. Cytochrome c: Using Biological Insight toward Engineering an Optimized Anticancer Biodrug. Inorganics 2021, 9 (11), 83 10.3390/inorganics9110083. 35978717 PMC9380692

[ref14] Geißler D.; Nirmalananthan-Budau N.; Scholtz L.; Tavernaro I.; Resch-Genger U. Analyzing the surface of functional nanomaterials—how to quantify the total and derivatizable number of functional groups and ligands. Microchimica Acta 2021, 188 (10), 321 10.1007/s00604-021-04960-5. 34482449 PMC8418596

[ref15] Ju S.; Yeo W.-S. Quantification of proteins on gold nanoparticles by combining MALDI-TOF MS and proteolysis. Nanotechnology 2012, 23 (13), 135701 10.1088/0957-4484/23/13/135701. 22417878

[ref16] Scolaro L. M.; Romeo A.; Castriciano M. A.; Micali N. Unusual optical properties of porphyrin fractal J-aggregates. Chem. Commun. 2005, 24, 3018–3020. 10.1039/b501083g. 15959570

[ref17] Siggel U.; Bindig U.; Endisch C.; Komatsu T.; Tsuchida E.; Voigt J.; Fuhrhop J.-H. Photophysical and photochemical properties of porphyrin aggregates. Berichte der Bunsengesellschaft für physikalische Chemie 1996, 100 (12), 2070–2075. 10.1002/bbpc.19961001225.

[ref18] Haiss W.; Thanh N. T. K.; Aveyard J.; Fernig D. G. Determination of Size and Concentration of Gold Nanoparticles from UV–Vis Spectra. Anal. Chem. 2007, 79 (11), 4215–4221. 10.1021/ac0702084. 17458937

[ref19] Amendola V.; Meneghetti M. Size Evaluation of Gold Nanoparticles by UV–vis Spectroscopy. J. Phys. Chem. C 2009, 113 (11), 4277–4285. 10.1021/jp8082425.

