# Supplementary material for: Molecular Surface Quantification of Multifunctionalized Gold Nanoparticles Using UV–Visible Absorption Spectroscopy Deconvolution
Source: Anal Chem. 2023 Aug 25;95(35):12998–3002. doi: 10.1021/acs.analchem.3c01649 (PMC10483462; doi:10.1021/acs.analchem.3c01649)
Supplement: Supplementary file 1 — ac3c01649_si_001.pdf [file ac3c01649_si_001.pdf]

Supporting Information

Molecular Surface Quantification of Multi-Functionalized Gold Nanoparticles Using UV-Vis Spectroscopy Deconvolution

Jordan C. Potts[a], Akhil Jain[b], David B. Amabilino[c], Frankie J. Rawson[b]* and Lluïsa Pérez-García[a,d,e]*

[a] Division of Advanced Materials and Healthcare Technologies, School of Pharmacy, University of Nottingham, Nottingham NG7 2RD, UK

[b] Bioelectronics Laboratory, Division of Regenerative Medicine and Cellular Therapies, School of Pharmacy, University of Nottingham, Biodiscovery Institute, Nottingham NG7 2RD, UK

[c] Institut de Ciència de Materials de Barcelona (ICMAB), CSIC, Carrer dels Til·lers, Campus Universitari, 08193 Cerdanyola del Vallès, Catalunya, Spain

[d] Departament de Farmacologia, Toxicologia i Química Terapèutica, Facultat de Farmàcia i Ciències de l'Alimentació, Universitat de Barcelona, 08028 Barcelona, Spain

[e] Institut de Nanociència i Nanotecnologia UB (IN2UB), Universitat de Barcelona, 08028 Barcelona, Spain

Frankie.Rawson@nottingham.ac.uk* and mlperez@ub.edu*

Methods.

Equine heart cytochrome *C* (Cyt *C*) (≥ 95%), *N*-(3-dimethylaminopropyl)-*N'*-ethylcarbodiimide hydrochloride (EDC), gold(III) chloride trihydrate ($HAuCl_4{\cdot}3H_2O$) (99.99%), thiol-PEG-carboxyl (HS-PEG-COOH) (2000 Da), 2-(*N*-morpholino)ethanesulfonic acid, 4-morpholineethanesulfonic acid monohydrate (MES monohydrate) (≥99.0%), *N*-hydroxysuccinimide (NHS) (98%), sodium citrate tribasic dihydrate (99%) were purchased from Sigma Aldrich. 5-(4-Aminophenyl)-10,15,20-tris-(4-sulfonatophenyl)porphyrin (Porph) and Zinc(II) 5-(4-aminophenyl)-10,15,20-tris-(4-sulfonatophenyl)porphyrin (Zn Porph) were purchased from Porphychem, France. Milli-Q® water at a resistivity of 18.2 MΩ provided by Milli-Q® direct water purification system was used for experiments.

Synthesis of 20 nm citrate capped AuNPs (cit-AuNPs)

1 mL of $HAuCl_4.3H_2O$ (39.38 mg, 0.1 mol) was added to 89 mL of Milli-Q® water and was brought to boil under constant stirring. Aqueous sodium citrate solution (38.8 mM, 10 mL) was rapidly added to the boiling solution causing a colour change from yellow to burgundy. The solution was boiled for 10 minutes before allowing it to cool down at room temperature under stirring. The solution was then filtered using a 0.22 µm nylon filter to remove any large aggregates and stored at 4°C until further use.

Synthesis of 50 nm citrate capped AuNPs (cit-AuNPs)

A total of 100 µL of $HAuCl_4.3H_2O$ (1% W/V) was added to a scintillation vial containing 9.488 mL of Milli-Q® water before the addition of 20 nm AuNPs (290 µL) which was then set to stir at room temperature for 10 minuets. 22 µL of Sodium citrate tribasic dihydrate (1% W/V) was then added to the stirring solution and immediately followed by the addition of 100 µL of Hydroquinone (30mM). The solution rapidly changed colour and was mixed for a further 10 minutes. The obtained coloured solution was filtered using 0.22 µm filter and stored at 4°C until further use.

Functionalization of 20 nm cit-AuNPs with PEG, Cyt C and Zn Porph

A 1 mL solution of aqueous HS-PEG-COOH (2000 Da, 1 mM) was added to 20 mL of 20 nm or 50 nm cit-AuNPs under stirring and left overnight. The following day the solution was washed twice using centrifugation at 13,400 rpm for 30 minutes to form a pellet of AuNP functionalized with PEG-COOH (AuNP-PEG). The pellet was then resuspended in ultrapure water and stored at 4°C until further use.

For covalent conjugation of Cyt *C* and Zn Porph on both 20 nm and 50 nm AuNP-PEG, a fresh EDC/NHS mix solution was made by adding EDC (19.17 mg, 100 µmol) and NHS (23 mg, 200 µmol) in 1 mL of MES buffer (10 mM, pH 5). A total of 100µL of EDC/NHS mix was added to the 1 mL aqueous solution of AuNP-PEG and was left stirring for 1 hour. The solution was then centrifuged at 13,400 rpm for 30 minutes to form a pellet of the AuNP with activated PEG-COOH. To this pellet aqueous Cyt *C* (1 mL, 200 µM) and aqueous Zn Porph (1 mL, 200 µM) were added under constant stirring. For the synthesis of AuNP-PEG-Cyt *C* only Cyt C (1 mL, 200 µM) was added, and in AuNP-PEG-Zn Porph only Zn Porph (1 mL, 200 µM) was added to the EDC/NHS activated AuNP-PEG . The solution was stirred for 24 hours followed by three cycles of centrifugation at 13,400 rpm using Milli-Q® water to remove any unbound Cyt *C* and Zn Porph. The obtained pellet was resuspended in 1 mL of Milli-Q® water to yield a monodispersed sample of AuNP-PEG-Cyt *C*/Zn Porph.

Deconvolution modelling of Cyt C and Zn Porph conjugated to AuNPs

Deconvolution of UV-visible absorption spectrum confirming the binding of Cyt *C* and Zn Porph was performed using CASA-XPS. The absorption spectra of the chosen samples were imported into CASA-XPS as text files. The graph was then plotted, and the x-axis was changed to start with the smallest number near the origin. The region of interest was then selected using the regions tool, and then the background setting was changed to 'linear' as it closely represents how the spectra would look without the conjugated molecules. The convoluted peaks in the spectra were then found using the components tab, by adding the components that were thought to be in the region. The components were then fitted to the spectra using Levenberg-Marquardt algorithm LN (LN-MIE-Gans) fitting. The resulting spectra data was copied to Microsoft Excel before being added to GraphPad Prism 9 for analysis.

LN-MIE-Gans modelling of cit-AuNPs and multi-functionalized AuNPs

Model fitting on AuNP spectra to predict their size was conducted on Wolfram Mathematica using the LNMG Fit 1.0 model obtained from the literature.[1]

Instruments

Ultraviolet-visible (UV-visible absorption) absorption spectra were obtained using a Varian Cary 50 bio-UV-visible absorption spectrophotometer. Centrifugation was performed on a Spectrafuge 24D. The transmission electron microscope (TEM) images were captured using JEOL FX 2000 TEM at 200 kV. The histogram of AuNP size distribution was determined from the TEM images using ImageJ® software. Dynamic light scattering (DLS) and Zeta potential experiments were conducted on a Malvern Zetasizer nano.

Characterization of Multifunctionalized AuNPs

The physical characterization of the multi-functionalized AuNPs (AuNP-PEG-Cyt C/Zn Porph) was conducted to enable quantification of the conjugated molecules - Cyt C and Zn Porph. We initially performed light scattering techniques to assess the change in hydrodynamic diameter (hd) and zeta potential of cit-AuNPs (synthesized to be 20 nm), AuNPs-PEG, and AuNP-PEG-Cyt C/Zn Porph samples. The length of the fully extended HS-PEG-COOH (2000 Da) is ca. 7.9 nm, while the diameter of Cyt C is ca. 3.4 nm, and the diameter of Zn Porph is around ca. 1.6 nm (calculated using ChemDraw3D).[2] The hd of cit-AuNPs, AuNP-PEG and AuNP PEG-Cyt C/Zn Porph were measured using DLS (Figure S1A). This analysis revealed a difference in the hd of the Cit-AuNPs from 19.6±1.0 nm (Polydispersity index (PDI)-0.301) to 33.54±0.50 nm (PDI 0.297) in AuNP-PEG and 38.9±1.1 nm (PDI 0.388) for AuNP-PEG-Cyt C/Zn Porph, suggesting successful conjugation. The obtained PDI values indicate polydispersity in the samples, which is a typical characteristic of smaller size particles, in this case are 20 nm AuNPs.[3] Zeta potential measurements are useful in providing insight into the change in surface chemistry by measuring the potential difference between the stationary fluid attached to the particle and the dispersion medium itself.[4] Therefore, the zeta potential was used to confirm the successful conjugation of molecules to AuNPs. The zeta potential measurement of cit-AuNPs was recorded to be -36.77±2.33 mV which is a slightly lower value of -30.77 ±1.4 mV for AuNP-PEG. Cyt C is known to have a positive zeta potential measurement which is exhibited on the AuNP-PEG-Cyt C samples at +14.7 ±0.9 mV, confirming the successful conjugation of Cyt C on PEG functionalized AuNPs (Figure S1B).[4] On the other hand, the zeta potential values of AuNP-PEG-Zn Porph at -27.3±0 mV vs AuNP-PEG indicate the successful conjugation of Zn Porph. The change in zeta potential values of -27.3 mV in AuNP-PEG-Zn Porph to +10.8 ±0.2 mV for AuNP-PEG-Cyt C /Zn Porph samples confirms the successful conjugation of both Cyt C and Zn Porph to AuNPs-PEG.TEM was carried out to analyze the morphology, aggregation, and size of surface functionalized 20 nm AuNPs. TEM analysis revealed that the AuNP-PEG-Cyt C /Zn Porph particles are monodisperse, and the functionalization process does not cause any aggregation (Figure S1C). Furthermore, the cumulative frequency graph (inset in Figure S1C) reveals a mean average diameter of AuNP-PEG- Cyt C /Zn Porph samples to be around 19.15±2.12 nm (Gaussian fit). The size discrepancy between TEM and DLS is because DLS measures the hd of particles in suspension while TEM images provide information about the particle size in the dry state. DLS can differentiate between the Cit-AuNPs and the multi-functionalized AuNPs as the hd differs due to the change in the organic layer surrounding the AuNPs, the contrast produced in TEM would not be great enough to determine the change in diameter .

To further confirm the successful conjugation of Cyt C and Zn Porph to AuNPs (20 nm), UV-visible absorption spectroscopy was carried out (Figure. 2D), which is utilized widely to characterize AuNPs and their surface functionalization.[5] The maximum absorbance (located between 500 and 600 nm) in a typical UV-Vis absorption spectrum of AuNPs, also known as the surface plasmon resonance (SPR) peak, is indicative of their size. Once the size and the extinction coefficient of the AuNPs are known, their concentration can be calculated using the Beer-Lambert law.[5, 6] On the other hand, porphyrins are commonly conjugated to nanomaterials for fluorescent tagging and photodynamic therapy. [7] The UV-visible absorption spectra of AuNPs synthesized in this work are reported in Figure S1D, which revealed that the SPR peak of the AuNPs remains in the same position at 521 nm for all the samples except AuNP-PEG-Cyt C, in which it has red-shifted to 525 nm compared to cit-AuNPs (521 nm), indicating a change in surface chemistry. This could be attributed to the successful functionalization of Cyt C onto AuNP-PEG. Moreover, full-width half maximum (FWHM) of the SPR peak increased in all the functionalized AuNP samples when compared to the spectrum of 20 nm cit-AuNPs, suggesting an increase in polydispersity of the functionalized AuNPs, which could be the result of agglomerates which could form in the colloidal dispersions. The increase in intensity between 650-700 nm in AuNP-PEG-Cyt C is likely to be the result of small aggregates caused by protein-nanoparticle interactions between Cyt C conjugated to AuNPs. Cit-AuNPs and AuNPs-PEG do not show any absorption peaks other than the SPR., whereas free Cyt C and Zn Porph exhibit additional peaks, and their UV-visible absorption spectra are shown in Figure S1A. The Soret band peaks at 408 nm and 415 nm are for oxidised (Cyt C Ox) and reduced Cyt C (Cyt C Red), respectively, which aligns with literature values.[8] The absorption peak at 423 nm corresponds to Zn Porph (Figure S2A). After conjugation to AuNPs, the Soret band peak of Cyt C was located at 410 nm, suggesting that the Cyt C conjugated to the AuNPs exists in both oxidized and reduced forms. While the Zn Porph Soret band is evident at 430 nm, which is red shifted when compared to free Zn Porph in solution at 423 nm. This red shift in Zn Porph peak after conjugation to AuNPs could be attributed to J-type aggregation formed between neighbouring chromophores (in this case Zn Porph) attached close to each other in a nanoparticle.[9] It is evident from Figure S1D that in multifunctional AuNP-PEG-Cyt C/Zn Porph samples, the Soret band peaks of Cyt C and Zn Porph overlap; consequently, the exact peak of each component is difficult to determine. Importantly, this poses the risk of erroneous calculations to determine the accurate concentrations of these molecules. The overlapping spectra of Cyt C Ox and Zn Porph (Figure S2B) and Cyt C Red and Zn Porph (Figure S2C) free in solution support the spectrum depicted in Figure S1D showing how the absorbance peaks convolute and make accurate peak determination impossible. Therefore, the deconvolution of the overlapping spectra of Cyt C Ox and Zn Porph and Cyt C Red and Zn Porph (Figure S2D and Figure S2E) is essential as it allows the hidden peaks to be revealed, and therefore the absorbance of each peak to be identified for further quantification purpose.

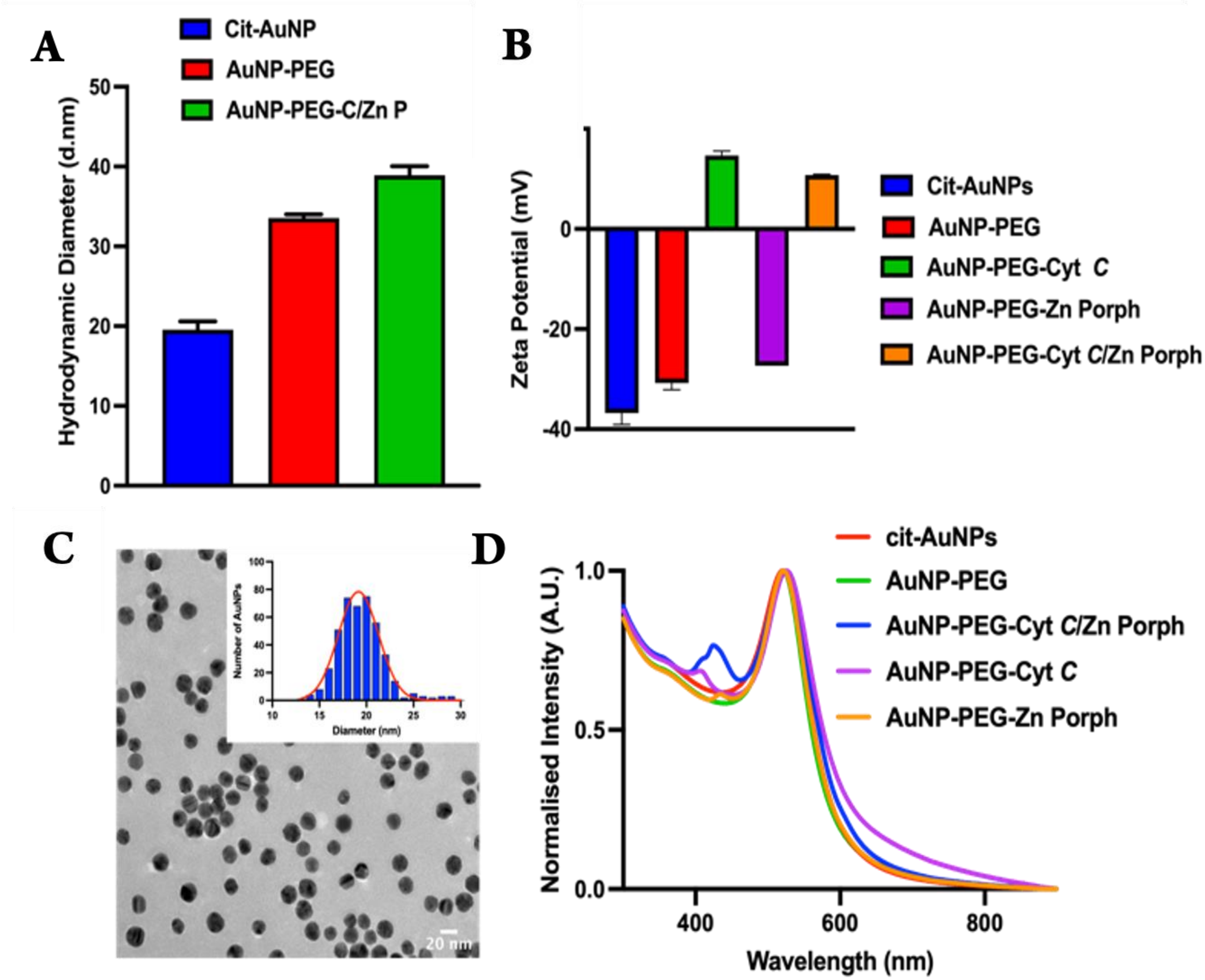

Figure S1. The physicochemical characterization of AuNPs functionalized with Cyt *C* and Zn Porph was performed using DLS(A), Zeta Potential measurements (B), TEM image, and cumulative frequency graph of 20 nm AuNP-PEG-Cyt *C* /Zn Porph produced from analysing 452 AuNPs (C) and UV-visible absorption spectroscopy (D).

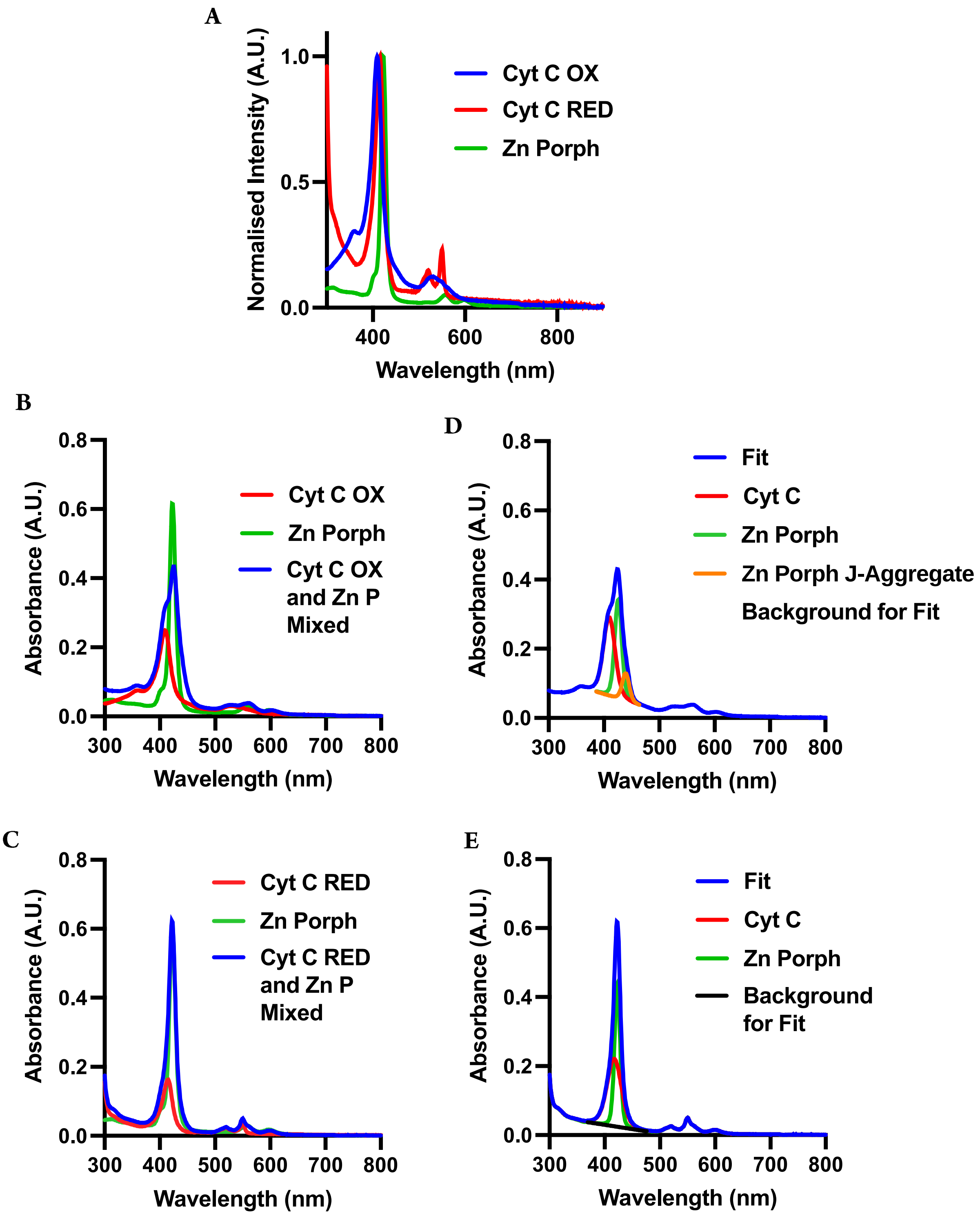

Figure S2. Ultraviolet-Visible absorption spectra of (**A**) Cyt C OX, Cyt C RED and Zn Porph free in solution with normalized intensity. (**B**) Cyt C OX and Zn Porph mixed and independently free in solution at 2.5 mM. (**C**) Cyt C RED and Zn Porph mixed and independently free in solution at 2.5 mM. (**D**) Deconvolution and fitted spectra of Cyt C OX and Zn Porph mixed to reveal hidden spectra of Cyt C and Zn Porph individual components. (**E**) Deconvolution and fitted spectra of Cyt C RED and Zn Porph mixed to reveal hidden spectra of Cyt C and Zn Porph individual components.

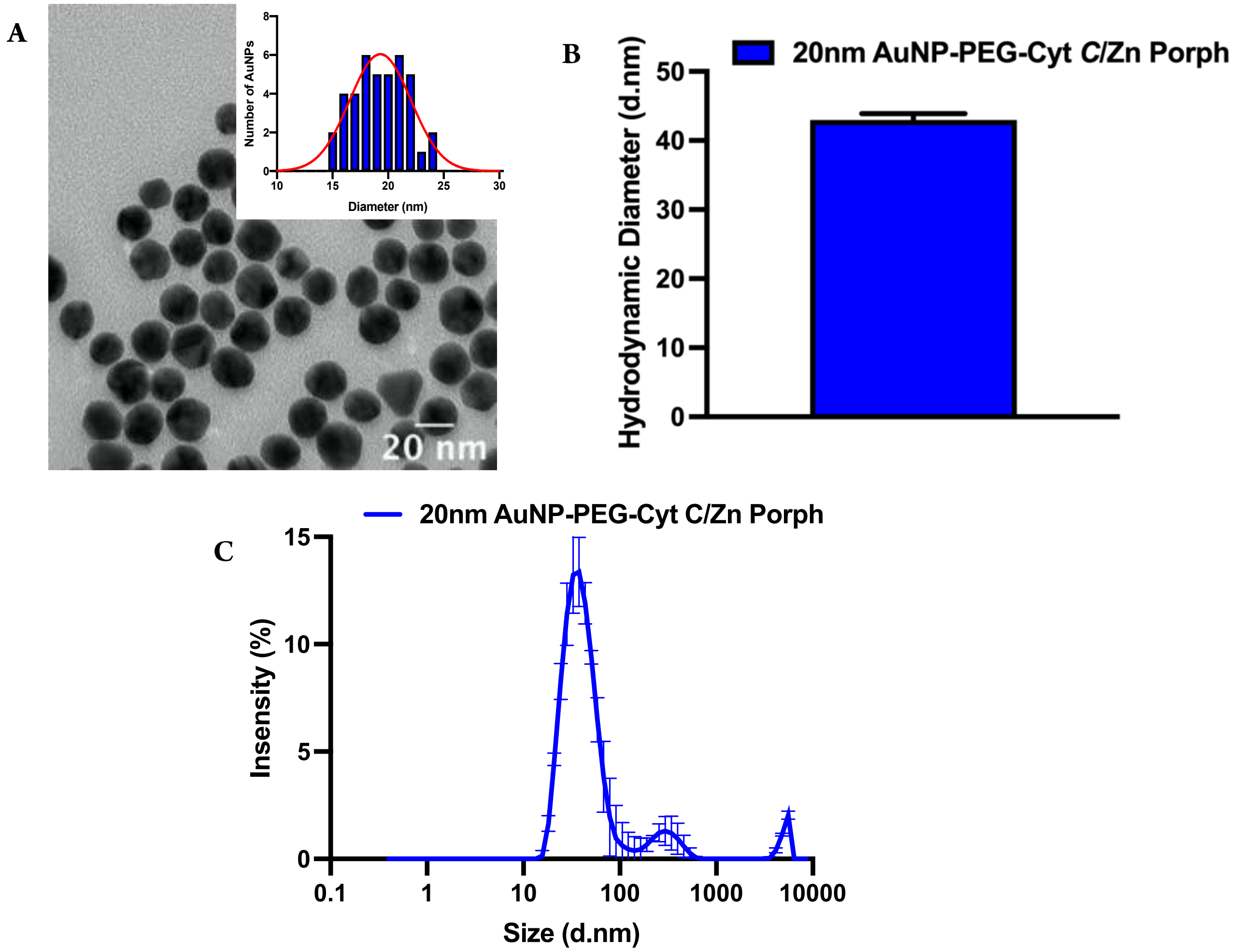

Figure S3. Physico-chemical characterization of 20 nm AuNP-PEG-Cyt C/Zn Porph-S2 from Figure 3B with TEM and cumulative frequency distribution calculated from 40 AuNPs (**A**), DLS average diameter (**B**) and DLS intensity profile (**C**)

Table S1. AuNP, Cyt *C* and Zn Porph concentration and number of molecules calculated from the UV-Vis absorption spectra of Figure 3A and 3B

	AuNP		Cyt C			Zn Porph/Porph			
**Sample**	Concentration (nM)	Number of Molecules	Concentration (μM)	Number of Molecules	Number Per AuNP	Concentration (μM)	Number of Molecules	No. of molecules Per AuNP	Free Zn Porph Concentration (μM)
**20 nm AuNP-PEG-Cyt C /Zn Porph (Figure 3A)**	1.21	$7.30x10^{14}$	0.42	$2.53x10^{17}$	346	1.30	$7.83x10^{17}$	1073	-
**20 nm AuNP-PEG-Cyt C/Zn Porph-S2 (Figure 3B)**	0.26	$1.55x10^{14}$	0.40	$2.40x10^{17}$	1545	1.16	$6.99x10^{17}$	4500	1.11
**50 nm AuNP-PEG-Cyt C/Porph(Figure S4A)**	0.0096	$5.79x10^{12}$	0.11	$6.73x10^{16}$	23259	0.17	$1.02x10^{17}$	35168	

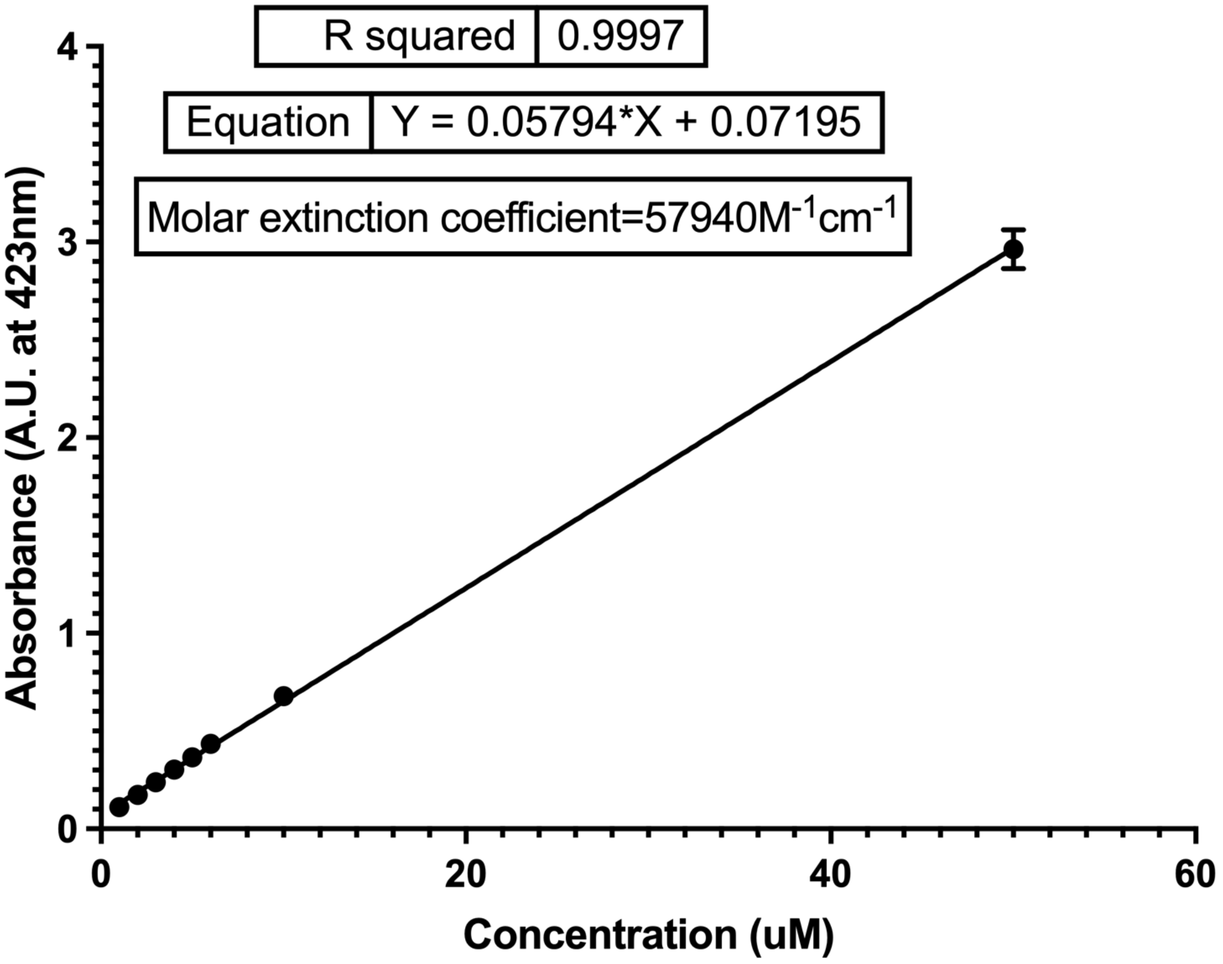

Figure S4. The concentration plot used to calculate the extinction coefficient of Zn Porph

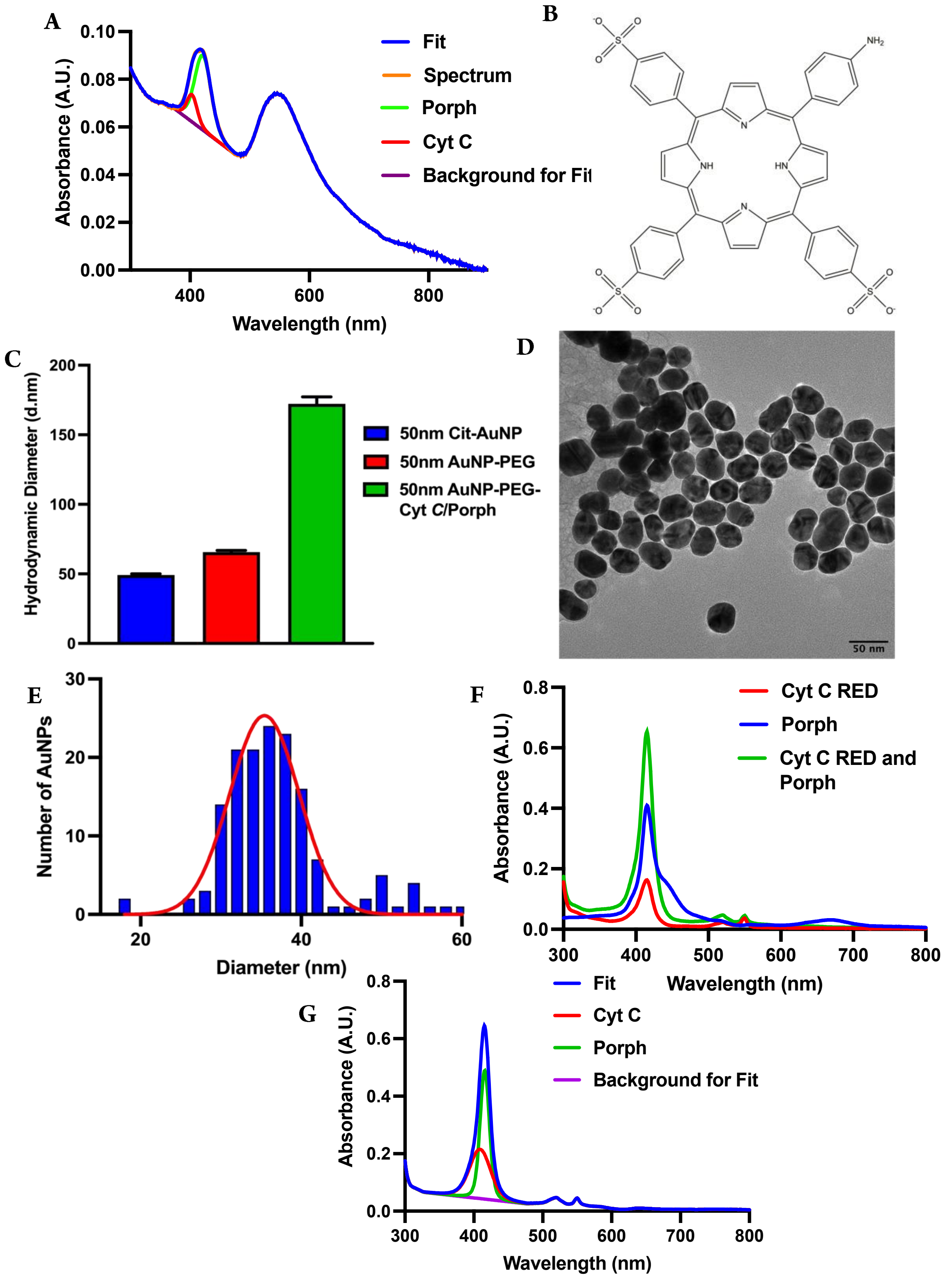

Figure S5. UV-Vis absorption spectrum of 50 nm AuNP-PEG-Cyt *C*/Porph with Soret band deconvolution **(A)**. Structure of Porph (extinction coefficient 184900 M^{-1} cm^{-1}) **(B)**. DLS of 50 nm Cit-AuNPs, 50 nm AuNP-PEG and 50 nm AuNP-PEG-Cyt *C*/Zn Porph **(C)**. TEM of 50 nm AuNP-PEG-Cyt *C*/Zn Porph **(D)**. Cumulative frequency distribution of 50 nm AuNP-PEG-Cyt *C*/Zn Porph calculated from 163 AuNPs **(E)**. UV-Vis absorption spectra of Cyt *C* RED, Porph and both mixed free in solution at 2.5μM **(F)**. Deconvolution of UV-Vis absorption spectrum of Cyt *C* RED and Porph mixed in solution **(G)**.

A

B

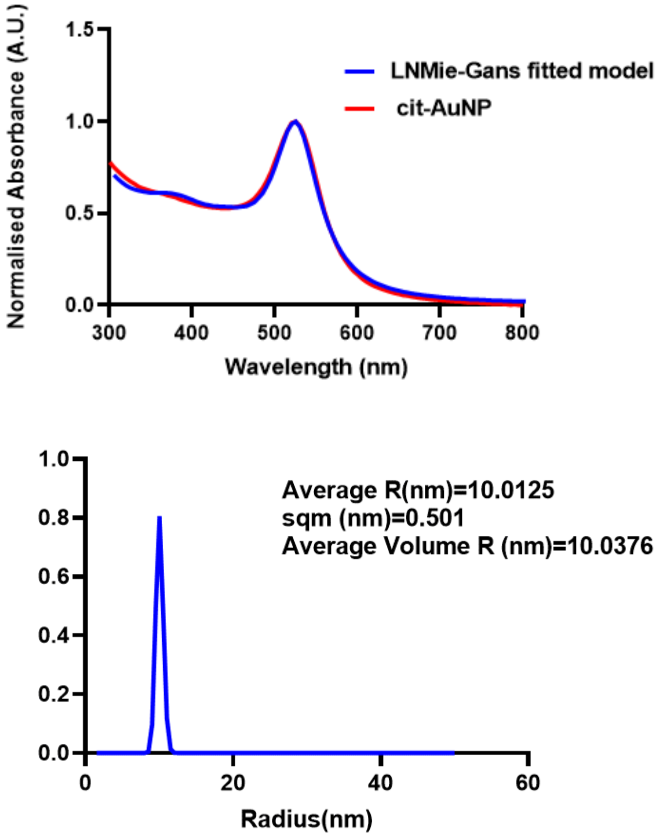

Figure S6. The use of LNMIE-Gans model fitting to 20 nm cit-AuNPs (A) to predict their size (B)

References:

(1) Amendola, V.; Meneghetti, M. Size Evaluation of Gold Nanoparticles by UV−vis Spectroscopy. *The Journal of Physical Chemistry C* **2009**, *113* (11), 4277-4285. DOI: 10.1021/jp8082425.
(2) Garrido, C.; Galluzzi, L.; Brunet, M.; Puig, P. E.; Didelot, C.; Kroemer, G. Mechanisms of cytochrome c release from mitochondria. *Cell Death And Differentiation* **2006**, *13*, 1423, Review. DOI: 10.1038/sj.cdd.4401950.
(3) Dheyab, M.; Abdul Aziz, A.; Jameel, M.; Moradi Khaniabadi, P.; Oglat, A. Rapid Sonochemically-Assisted Synthesis of Highly Stable Gold Nanoparticles as Computed Tomography Contrast Agents. *Applied Sciences* **2020**, *10*, 7020. DOI: 10.3390/app10207020.
(4) Jain, A.; Trindade, G. F.; Hicks, J. M.; Potts, J. C.; Rahman, R.; Hague, R. J. M.; Amabilino, D. B.; Pérez-García, L.; Rawson, F. J. Modulating the biological function of protein by tailoring the adsorption orientation on nanoparticles. *Journal of Colloid and Interface Science* **2021**, *587*, 150-161. DOI: https://doi.org/10.1016/j.jcis.2020.12.025.
(5) Haiss, W.; Thanh, N. T. K.; Aveyard, J.; Fernig, D. G. Determination of Size and Concentration of Gold Nanoparticles from UV−Vis Spectra. *Analytical Chemistry* **2007**, *79* (11), 4215-4221. DOI: 10.1021/ac0702084.
(6) Grimsley, G. R.; Pace, C. N. Spectrophotometric determination of protein concentration. *Curr Protoc Protein Sci* **2004**, *Chapter 3*, Unit 3.1. DOI: 10.1002/0471140864.ps0301s33 From NLM. Fonin, A. V.; Sulatskaya, A. I.; Kuznetsova, I. M.; Turoverov, K. K. Fluorescence of dyes in solutions with high absorbance. Inner filter effect correction. *PloS one* **2014**, *9* (7), e103878-e103878.

(7) Tsolekile, N.; Nahle, S.; Zikalala, N.; Parani, S.; Sakho, E. H. M.; Joubert, O.; Matoetoe, M. C.; Songca, S. P.; Oluwafemi, O. S. Cytotoxicity, fluorescence tagging and gene-expression study of CuInS/ZnS QDS - meso (hydroxyphenyl) porphyrin conjugate against human monocytic leukemia cells. *Scientific Reports* **2020**, *10* (1), 4936. DOI: 10.1038/s41598-020-61881-8. Kou, J.; Dou, D.; Yang, L. Porphyrin photosensitizers in photodynamic therapy and its applications. *Oncotarget* **2017**, *8* (46), 81591-81603. DOI: 10.18632/oncotarget.20189 PubMed. Sharma, B.; Jain, A.; Pérez-García, L.; Watts, J. A.; Rawson, F. J.; Chaudhary, G. R.; Kaur, G. Metallocatanionic vesicle-mediated enhanced singlet oxygen generation and photodynamic therapy of cancer cells. *Journal of Materials Chemistry B* **2022**, *10* (13), 2160-2170, 10.1039/D2TB00011C. DOI: 10.1039/D2TB00011C. García Calavia, P.; Bruce, G.; Pérez-García, L.; Russell, D. A. Photosensitiser-gold nanoparticle conjugates for photodynamic therapy of cancer. *Photochem Photobiol Sci* **2018**, *17* (11), 1534-1552. DOI: 10.1039/c8pp00271a From NLM.
(8) Fisher, W. R.; Taniuchi, H.; Anfinsen, C. B. On the Role of Heme in the Formation of the Structure of Cytochrome c. *Journal of Biological Chemistry* **1973**, *248* (9), 3188-3195. DOI: https://doi.org/10.1016/S0021-9258(19)44026-X.
(9) Maiti, N. C.; Mazumdar, S.; Periasamy, N. J- and H-Aggregates of Porphyrin−Surfactant Complexes: Time-Resolved Fluorescence and Other Spectroscopic Studies. *The Journal of Physical Chemistry B* **1998**, *102* (9), 1528-1538.
